# Neural Oscillation Associated with Contagious Itch in Patients with Atopic Dermatitis

**DOI:** 10.3390/brainsci11040438

**Published:** 2021-03-29

**Authors:** In-Seon Lee, Kyuseok Kim, Hi-Joon Park, Hyangsook Lee, Won-Mo Jung, Do-Won Kim, Younbyoung Chae

**Affiliations:** 1 Acupuncture and Meridian Science Research Center, Kyung Hee University, Seoul 02447, Korea; inseon.lee@khu.ac.kr (I.-S.L.); acufind@khu.ac.kr (H.-J.P.); erc633@khu.ac.kr (H.L.); jungcro@gmail.com (W.-M.J.); 2 Department of Ophthalmology, Otorhinolaryngology and Dermatology of Korean Medicine, College of Korean Medicine, Kyung Hee University, Seoul 02447, Korea; kmdkskim@khu.ac.kr; 3 Department of Biomedical Engineering, Chonnam National University, Yeosu 59626, Korea

**Keywords:** atopic dermatitis, contagious itch, electroencephalography, mu rhythm, time–frequency analysis

## Abstract

Objective: Itch is an unpleasant sensation associated with an urge to scratch and is a major health care issue associated with atopic dermatitis (AD). Contagious itch, i.e., subjective feelings of itchiness induced by watching others’ scratching behavior, is common in patients with AD. Using electroencephalography, we examined alpha (8–13 Hz) oscillations in sensorimotor areas associated with the desire to scratch in patients with AD. Methods: Thirty-six patients with AD and 34 healthy controls (HCs) participated in this study. They evaluated their itch levels after watching short videos of a model scratching or tapping parts of his body. Neural oscillations were recorded from nine electrodes, including those placed over sensorimotor areas. Time–frequency analysis was used to compare mu rhythm suppression over the sensorimotor areas in response to these videos between patients with AD and HCs. Results: The behavioral test showed that the visual stimuli induced increased feelings of itchiness in patients with AD relative to HCs under the tapping and scratching conditions. The time–frequency analysis revealed that mu rhythm suppression in response to scratching images was significantly prominent in patients with AD, but not in HCs. Conclusion: Patients with AD exhibited increased susceptibility to contagious itch. This phenomenon might be related to enhanced mu rhythm suppression in sensorimotor areas of the brain in these patients. Our findings provide new insight into the neurophysiological basis of itch sensations in patients with AD.

## 1. Introduction

Itch is typically defined as “an unpleasant sensation associated with the desire to scratch” [1]. Itch has been known to exert evolutionary protective functions. However, patients with atopic dermatitis (AD) often struggle to resist the strong urge to scratch, in an uncontrollable vicious itch–scratch cycle [2]. Neuroimaging studies of itch processing have shown involvement of a network of brain regions, the so-called “itch matrix,” including sensory, motor, and affective components of the multidimensional itch sensation [3,4,5], which shares similarities with the pain processing network [6,7]. Recent meta-analyses on central mechanisms of itch revealed that the basal ganglia were commonly activated in patients with AD [8], whereas the thalamus, anterior insula, and frontal operculum were consistently activated across experimentally induced itch in healthy individuals [8,9]. As the corticostriatal circuit plays an important role in scratching behavior, this circuit may become a target for the inhibition of the excessive and habitual scratching behavior seen in patients with AD [10]. The results suggest that distinct neural mechanisms in the corticostriatal circuit associated with itch sensations and scratching behavior are active in patients with AD.

Contagious itch is a subjective feeling of itchiness induced by watching others’ scratching behavior [11,12]. The neural substrates mediating contagious itch are similar to the regions associated with histamine-induced itch [3,12,13,14]. For example, in healthy participants, observing others’ scratching movements activated the major brain areas of the itch matrix, including the thalamus, primary somatosensory cortex (SI), premotor cortex, and insula [15]. When healthy participants were shown itch-related or pain-related pictures, motor-related areas, including the supplementary motor area (SMA) and basal ganglia, as well as the anterior insula, were activated, with significantly greater functional connectivity observed between the anterior insula and motor areas during itch imagery relative to pain imagery [16]. 

Given that patients with AD tend to scratch themselves more often due to the itch–scratch cycle, we can expect that they might have greater behavioral and brain involvement in the planning of complex movements in response to contagious itch than do healthy controls (HCs). In previous studies, patients with AD showed more prominent itch-related responses to visual itch cues than did HCs [11,17]. Schut et al. [18] used functional magnetic resonance imaging (fMRI) to investigate the central mechanisms underlying contagious itch, and found that patients with AD showed greater brain activation in the frontostriatal circuits, including the SMA, ventral striatum, and orbitofrontal cortex, which play important roles in motivation, motor control, and preparation. Although viewing itch in others provokes motor reactions (scratching behavior) and functional brain activity in motor-related regions, whether motor-related neural responses are evoked differently or similarly in patients with AD remains unclear.

A lower amplitude of spontaneous brain oscillation can be associated with activation of a specific system [19]. For example, strong suppression of the mu rhythm, the central Rolandic or sensorimotor rhythm (frequency ranges of 8–13 Hz), which has been observed over the SI [20], has been shown to be associated with the perception of pain [21,22]. Furthermore, the perception of pain in others suppressed somatosensory oscillations consistent with the involvement of the mirror-neuron system [23]. Suppression of mu rhythm activity was also found during action execution and observation of action, as well as during imagined movement [20,24]. Recently, brain–computer interface systems based on event-related desynchronization (ERD) have been applied widely to generate induced activity by imagining motor movements [25]. Several brain regions associated with contagious itch, including the SI and premotor cortex, are related to simulated actions (mirror systems). Sensorimotor areas are important in the generation of an adequate motor response to itching stimuli in relation to the processed sensory information. Accordingly, we hypothesized that suppression of the mu rhythm may be a reliable indicator of the sensorimotor cortical resonance of contagious itch, especially in patients with AD. 

In the present study, we used electroencephalography (EEG) to examine the neurophysiological basis of contagious itch by measuring mu rhythm suppression over sensorimotor areas and comparing mu rhythm suppression between patients with AD and HCs.

## 2. Materials and Methods

### 2.1. Participants

Thirty-six patients with AD (21 females, age = 23.7 ± 0.8 years) and 34 healthy volunteers (24 females, age = 23.0 ± 0.6 years) were recruited for this study. All participants were neurologically healthy and had no other skin disease. Patients were recruited by print and email advertisements, as well as by physicians in the Department of Dermatology at Kyung Hee University Hospital. Patient eligibility was confirmed by a licensed dermatologist. The severity of AD was measured using the SCORing Atopic Dermatitis (SCORAD) index, and patients with mild-to-moderate AD were recruited for this study. Before the experiments, all participants provided state pruritus ratings (scale of 0–100; 0 = no itch at all, 100 = worst imaginable itch). All participants gave informed consent, and the protocol was approved by the Human Research Committee of Kyung Hee University Hospital.

### 2.2. Experimental Design and Procedures

**Figure 1:**
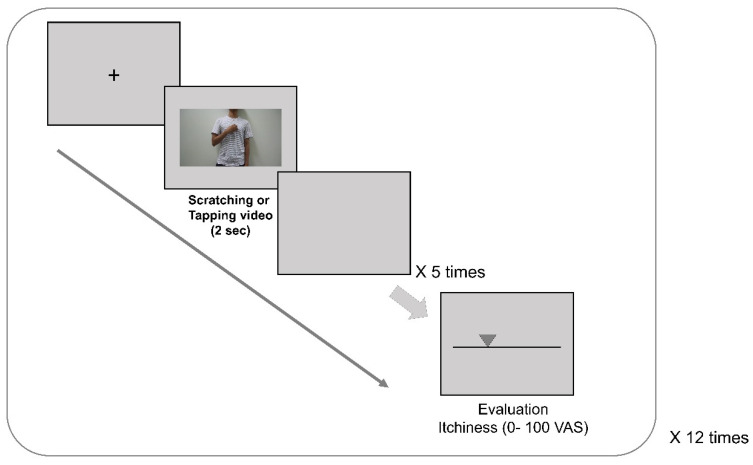
Experimental procedures for contagious itch induction. Thirty control (tapping behavior) videos were matched with 30 experimental (scratching behavior) videos. The scratching videos consisted of continuous scratching of the body sites (including the forearm, upper arm, chest, and neck areas) using four fingers of one hand. The control videos consisted of continuous tapping of the body sites. After viewing five consecutive scratching or tapping videos, participants were asked to rate their itch sensations (scale of 0–100; 0 = no itch at all, 100 = worst imaginable itch) using a response button.

Sixty 2 s video clips were created in advance for this experiment (Figure 1). Thirty control videos of a male tapping his fingers on his body were matched with 30 experimental videos of the same model scratching his body. The scratching behavior consisted of continuous scratching of body sites, such as the forearm, upper arm, chest, and neck, using four fingers of one hand. The tapping behavior consisted of continuous tapping of the same body parts. The model in the videos was filmed from the waist to the neck without showing his head or face. 

Participants were seated approximately 90 cm away from a computer screen in a quiet, soundproofed laboratory. They were asked to relax as much as possible while watching the videos without moving their heads or bodies. All participants were instructed not to scratch their bodies during the experiment. After watching five consecutive scratching or tapping videos (total of 10 s), they were asked to rate their itch levels (scale of 0–100; 0 = no itch at all, 100 = worst imaginable itch) using a response button. 

### 2.3. EEG Recording and Analysis

Electrodes were positioned according to the extended 10–20 system, and electrical activities were recorded at 1000 Hz using BrainVision actiCHamp (Brain Products, Munich, Germany) and BrainVision Recorder software (version 1.21.0303, Brain Products). Data were collected from 10 electrodes at the following scalp positions: Fp1, Fp2, F3, F4, C3, Cz, C4, P3, P4, and Fz (reference). Electrode impedance was maintained at <5 KΩ. Raw EEG signals were amplified with band pass (1–30 Hz) and then filtered with a 50 Hz notch filter. Filtered EEG signals were preprocessed using the BrainVision Analyzer software (version 2.2, Brain Products). EEG data were down-sampled at 250 Hz and filtered with a high-pass filter at 1 Hz. Vertical and horizontal eye movements were corrected by principal component analysis (PCA). 

Offline analyses were conducted with MATLAB (MathWorks, Natick, MA, USA) scripts using functions from the EEGLAB environment [26]. Time–frequency analysis was performed using Fourier transform implemented in EEGLAB. Time–frequency transformation was performed for frequencies up to 30 Hz in a time window extending from −500 to 1500 ms with respect to the onset of visual stimulation, resulting in a 2000 ms window for each epoch. Baseline correction was performed using the pre-onset epochs (−500 to 0 ms). Epochs with non-stereotyped artifacts were excluded. Data from participants who completed less than 50% of the trials were not included in the analysis to protect the overall quality of the data. 

ERD was estimated as the change in the frequency band power of the mu rhythm (8–13 Hz) recorded on channels located over the left and right sensorimotor cortices (C3 and C4, respectively). Although data were obtained from all electrodes across the scalp, mu suppression was defined as the mean power measured over the sensorimotor cortex (C3, Cz, and C4) [27,28]. As a control for mu suppression, the mean amplitude of power changes in the alpha band (8–13 Hz) during the time window up to 1500 ms was determined. Neurophysiological changes related to contagious itch were compared by measuring the mu suppression effects between scratching and tapping conditions.

### 2.4. Statistical Analysis

All results are expressed as means ± standard errors. The data were analyzed using a 2 × 2 analysis of variance (ANOVA), with the type of video stimulus (tapping or scratching) serving as the within factor and group (AD or HC) serving as the between factor. Pairwise comparison tests were Bonferroni corrected, and the alpha level was set at *p* < 0.05.

## 3. Results

### 3.1. Participants

The SCORAD score for patients with AD was 34.6 ± 1.5. Before the experiments, pruritus ratings were significantly greater among patients with AD than among HCs (6.1 ± 0.2 vs. 1.7 ± 0.3; *t* = 14.6, *p* < 0.001).

### 3.2. Contagious Itch Ratings in Response to Visual Stimuli 

**Figure 2:**
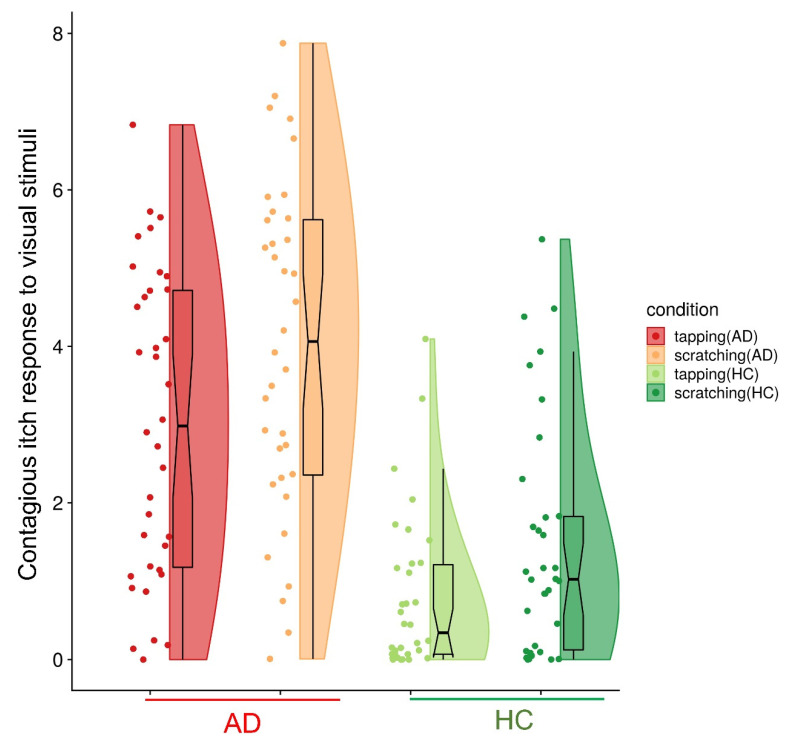
Contagious itch responses in patients with atopic dermatitis (AD) and healthy controls (HCs). Patients with AD and HCs reported significantly higher itch ratings in response to the scratching videos than to the tapping videos (AD: tapping = 3.01 ± 0.33, scratching = 4.00 ± 0.36; HC: tapping = 0.78 ± 0.18, scratching = 1.46 ± 0.26).

Patients with AD and HCs reported significantly higher itch ratings in response to the scratching than in response to the tapping videos (AD: tapping = 3.01 ± 0.33, scratching = 4.00 ± 0.36; HC: tapping = 0.78 ± 0.18, scratching = 1.46 ± 0.26; Figure 2). ANOVA revealed significant main effects of video type (F = 34.62, *p* < 0.001) and group (F = 38.90, *p* < 0.001); the interaction effect (video type × group) was not significant (F = 1.14, *p* = 0.290). 

### 3.3. Mu Suppression during Contagious Itch

**Figure 3:**
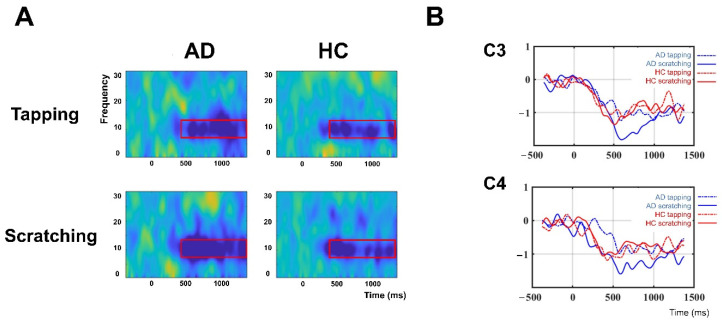
Mu suppression during contagious itch. (**A**): Averaged time–frequency responses in the left sensorimotor area (C3). The red box represents the time–frequency window of interest in the current study (mu rhythm 8–13 Hz). (**B**): Group mean locations and time courses of the somatosensory oscillations associated with contagious itch. Mean event-related desynchronization in the frequency range of 8–13 Hz was compared between the atopic dermatitis (AD, indicated by blue line) and healthy control (HC, indicated by red line) groups, and between the visual stimuli (tapping, indicated by a dotted line, and scratching, indicated by a solid line). Mu rhythm suppression in response to scratching videos was significantly increased in patients with AD, but not in HCs.

The time–frequency analysis revealed significantly prominent mu rhythm suppression during scratching videos in patients with AD, but not in HCs. ANOVA revealed a significant interaction effect (video type × group; F = 7.40, *p* < 0.01). Post hoc analysis revealed a significant main effect of video type in the AD group (F = 12.39, *p* < 0.001), but not in the HC group (F = 0.02, *p* = 0.902; Figure 3).

## 4. Discussion

We found that patients with AD exhibited greater susceptibility to contagious itch than did healthy subjects, consistent with previous results. In addition, we demonstrated that suppression of the mu rhythm during contagious itch was significantly enhanced in patients with AD relative to healthy subjects. Our findings suggest that brain oscillations in sensorimotor areas are involved in contagious itch in patients with AD, providing new insight into the neurophysiological basis of chronic itch in these patients. 

In the current study, patients with AD and healthy participants showed increased itch sensations in response to scratching videos relative to tapping videos. This result corresponds with previous findings of increased itch while watching short video clips of other people scratching relative to watching a control video [11,17]. Similar to the principle of classical conditioning, scratching cues may act as conditioned stimuli and lead to greater scratch responses in patients with AD than in HCs [12]. For example, patients with chronic itch showed greater conditioned scratch responses than did HCs [29,30]. As patients with AD are prone to feel more itch while watching others’ scratching behavior, an enhanced urge to scratch in response to itch-related cues should be considered to be a clinically significant impairment, along with allergen-induced pruritus, in patients with AD. Furthermore, an understanding of the psychosocial characteristics of itch sensations in patients with AD would improve current itch management strategies. Since the itch intensity can be enhanced by watching other’s scratching behaviors, it will be important to prevent chronic patients with AD from feeling contagious itch, for example, sharing a room with each other in the hospital.

This study demonstrated that patients with chronic pruritus had central sensitization to itch sensations [31]. As the corticostriatal circuit plays an important role in scratching behavior, this circuit may be an effective target for the inhibition of excessive and habitual scratching behavior in patients with AD [10]. A few neuroimaging studies have shown distinct motor-related neural patterns in patients with AD compared with HCs. Schneider et al. [32] showed significantly greater activity in the basal ganglia, which are involved in motor control, the motivation to act, and craving, in patients with AD than in HCs during histamine-induced itch. Schut et al. [18] demonstrated that the viewing of scratching videos can evoke brain activation in the frontostriatal circuits, which are involved in motivation, motor control, and preparation, in patients with AD. However, neural changes during contagious itch have not been compared directly between patients with AD and HCs. Scratching can damage the skin and aggravate itch symptoms, making contagious itch a significant problem for patients with AD. We found that the greatest mu suppression among the corticostriatal circuits while watching scratching videos was in sensorimotor areas in patients with AD compared to HCs. 

Patients with AD and healthy controls exhibited prominent suppression of mu rhythms in response to the simulated actions (tapping and scratching). Especially, patients with AD exhibited increased susceptibility to contagious itch relative to healthy subjects, through enhanced mu suppression in sensorimotor areas of the brain. These results correspond with those of previous vicarious pain studies, in which mu suppression in the sensorimotor cortex and mirror neurons was prominent when participants perceived other people in painful situations [23,33]. These changes in neural oscillation are associated with activation of the sensorimotor cortex and the mirror neuron system, implicated in the simulation of others’ actions and feelings [20,34]. A feeling-based mirror system plays a crucial role in contagious itch, as the insula showed more sustained activity while participants watched scratching videos than while they watched tapping videos [15]. As we ruled out the effects of simple simulation of others’ actions by comparing the scratching condition with a tapping condition, we can conclude that the mirror system is more sensitive to itch-related information in patients with AD, and that brain oscillations in sensorimotor areas among corticostriatal circuits are involved in contagious itch in these patients. 

This study has some limitations that should be addressed. First, we analyzed mu suppression only over the sensorimotor cortex, where it is prominent, and not in other brain areas. Previous fMRI studies using histamine-induced itch models were constrained by methodological limitations due to the rapid increase and slow decrease in itch sensations. EEG has limited spatial resolution; however, we applied time–frequency analysis to reveal brain oscillations during contagious itch in patients with AD. Second, due to the limited number of stimuli, we were not able to investigate the effect of body part (i.e., where the model tapped or scratched) on mu suppression during contagious itch, although the videos showed scratching and tapping of various parts of the body. Moreover, we used a single male model in the videos, which might have affected participants’ perception of his motions. In future research, we will investigate the effect of body part, including whether a body part shows atopic skin lesions, and the effect of using various models in the videos. Third, we did not measure personality or empathy. As brain activities in response to contagious itch can vary with psychological factors, including neuroticism [15], psychological factors should be considered in future studies.

## 5. Conclusions

In summary, patients with AD were more vulnerable to contagious itch than were HCs, and the contagious itch phenomenon might be derived from enhanced mu rhythm suppression in the sensorimotor area of the brain. Our findings provide a clue to the understanding of neural and behavioral itch responses and might lead to the development of new strategies or treatments for itch sensations in patients with AD.

## Data Availability

Data available on request from the authors.
